# Impact of Completeness of Revascularization on Long-Term Outcomes in Patients With Post-Infarction Ventricular Septal Rupture

**DOI:** 10.31083/RCM27049

**Published:** 2025-06-16

**Authors:** Jiexu Ma, Hang Xu, Shanshan Zheng, Zhiyuan Zhu, Sheng Liu

**Affiliations:** ^1^Department of Cardiovascular Surgery, Fuwai Hospital, State Key Laboratory of Cardiovascular Disease of China, National Center for Cardiovascular Diseases, Chinese Academy of Medical Sciences and Peking Union Medical College, 100037 Beijing, China

**Keywords:** myocardial infarction, revascularization, ventricular septal rupture, coronary artery bypass grafting

## Abstract

**Background:**

Ventricular septal rupture (VSR) is a life-threatening complication of myocardial infarction. While surgical repair is regarded as the definitive treatment, the optimal approach to revascularization remains uncertain. This study aims to evaluate the effects of infarct-related artery (IRA) revascularization and the completeness of revascularization on long-term survival and the incidence of major adverse cardiovascular and cerebrovascular events (MACCE) in patients with VSR.

**Methods:**

This retrospective study analyzed 132 VSR patients who underwent surgical repair at the Fuwai Hospital from 2004 to 2022. Patients were categorized based on whether they received IRA revascularization. For those with multi-vessel disease (MVD), revascularization was classified as complete or incomplete. The primary outcome was all-cause mortality, with a mean follow-up of 77.8 months (median 71.0 months). The secondary outcome was MACCE.

**Results:**

Of the 132 patients, 28 did not undergo IRA revascularization. Kaplan-Meier analysis showed similar all-cause mortality and MACCE rates between patients with and without IRA revascularization. Adjusted Cox regression confirmed no significant association between IRA revascularization and long-term mortality (adjusted hazard ratio [aHR], 0.62; 95% CI: 0.22–1.79) or MACCE (aHR, 1.30; 95% CI: 0.52–3.27). These findings were consistent across both single-vessel and MVD patients. Among the 84 MVD patients, 53 underwent complete revascularization. Patients with complete revascularization had a lower incidence of MACCE (aHR, 0.26; 95% CI: 0.10–0.67) compared to those with incomplete revascularization, although no significant difference in mortality was observed (aHR, 0.57; 95% CI: 0.17–1.85).

**Conclusions:**

IRA revascularization does not affect long-term survival or MACCE rates in VSR patients. However, complete revascularization significantly reduces the risk of MACCE in patients with MVD.

## 1. Introduction

Ventricular septal rupture (VSR) is a rare but life-threatening mechanical complication of acute myocardial infarction (AMI) [1]. While surgical repair is widely regarded as the definitive treatment, the role of revascularization—a cornerstone of coronary artery disease (CAD) management—remains uncertain and is less commonly performed in VSR patients compared to those without mechanical complications [2, 3].

A key debate in VSR management revolves around the benefits of infarct-related artery (IRA) revascularization [4, 5, 6]. Since VSR arises from coronary artery occlusion, revascularization appears to be a logical therapeutic strategy [7, 8]. However, the occurrence of VSR reflects excessive ischemia-induced oxidative stress, cytokine release, and activation of matrix metalloproteinases, which severely degrade the extracellular matrix [9]. By the time VSR develops, the affected myocardium is often irreversibly necrotic, with significantly reduced oxygen consumption, raising doubts about the potential benefit of reperfusion [2]. Furthermore, even if IRA revascularization restores some myocardial function, its potential benefits may be modest and insufficient to outweigh the procedural risks associated with coronary artery bypass grafting (CABG) or percutaneous coronary intervention (PCI) [5, 10]. These concerns are particularly pronounced in patients with single-vessel disease, where the absence of coronary collateral circulation can isolate the affected myocardium and further limit the efficacy of revascularization [2, 4].

Another unresolved question concerns the role of revascularizing non-IRA in patients with multi-vessel disease (MVD). Advocates of complete revascularization (CR) argue that it may reduce the risk of recurrent ischemia and help control ventricular arrhythmias in the acute phase [5, 11]. However, the added complexity of CR, including prolonged cardiopulmonary bypass, may increase the risk of complications and early mortality, especially in unstable patients undergoing emergency procedures [4, 12, 13, 14].

Despite its established role in CAD management, the effectiveness of revascularization in VSR patients remains uncertain due to inconsistent findings in the literature. Furthermore, many studies fail to stratify patients by the extent of CAD, complicating comparisons between different revascularization strategies in well-matched cohorts [3, 6]. To address these knowledge gaps, we conducted a single-center observational study to evaluate the clinical significance of IRA revascularization and assess whether CR improves long-term outcomes in patients with MVD.

## 2. Materials and Methods

### 2.1 Study Population and Design

A total of 148 consecutive patients diagnosed with VSR who underwent surgical repair at the Fuwai Hospital between 2004 and 2022 were included in this single-center, retrospective cohort study. Patients were excluded if they experienced in-hospital mortality or death within 30 days post-surgery (n = 9), were lost to follow-up (n = 6), or had non-obstructive CAD (n = 1).

The relationship between IRA revascularization and long-term outcomes was investigated by comparing patients who received IRA revascularization with those who did not. Additionally, the prognostic impact of complete versus incomplete revascularization (ICR) was analyzed in patients with MVD. The Institutional Review Board of Fuwai Hospital approved the study (approval number: 2023-2139), and all participants provided informed consent.

### 2.2 Data Collection

All data were collected by experienced clinical researchers, and clinical definitions were applied in accordance with the 2013 American College of Cardiology Foundation/American Heart Association Key Data Elements and Definitions for CAD [15]. Patient demographics, cardiovascular risk factors, treatments, and imaging results were obtained from electronic medical records. Revascularization-related data—including the extent of CAD, culprit vessels, degree of luminal stenosis, treatment procedures (PCI or CABG), graft materials, and the number of anastomoses—were gathered from coronary angiography and surgical records. Follow-up was conducted through routine visits or telephone interviews by research staff using standardized forms and procedures.

### 2.3 Definitions and Outcomes

Coronary angiography was performed on all patients to visually estimate the degree of diameter stenosis. MVD was defined as 70% or greater luminal stenosis in two or more major epicardial arteries, or at least 50% stenosis in the left main trunk [8, 16]. Diffuse CAD was defined as significant stenosis with a length exceeding 20 mm [7]. The IRA was identified by integrating coronary angiography findings, the infarct territory, and the location of the septal rupture. Patients who underwent preoperative PCI or CABG during surgical repair to revascularize the IRA were included in the IRA revascularization group. In patients with MVD, CR was defined as revascularization of all significantly diseased major coronary arteries, either through bypass grafting or PCI [17, 18]. Given the variability in definitions of CR, we also examined associations between different stenosis severity thresholds (≥50% and ≥70%) and clinical outcomes [17].

The primary outcome was all-cause mortality, defined as death from any cause during the follow-up period. The secondary outcome was a composite of major adverse cardiovascular and cerebrovascular events (MACCE), including all-cause mortality, myocardial infarction, stroke, repeat revascularization, and readmission for acute coronary syndrome or heart failure.

### 2.4 Statistical Analysis

Continuous variables are presented as means ± standard deviations or medians with interquartile ranges (IQRs), while categorical variables are reported as frequencies and percentages. Group comparisons for continuous variables were performed using the independent *t*-test or the Mann-Whitney U test, and for categorical variables, the χ^2^ test or Fisher’s exact test, as appropriate.

Survival time was calculated from the date of discharge to the date of adverse events or the last follow-up visit. Cumulative event rates for different revascularization patterns were estimated using the Kaplan-Meier method and compared with the log-rank test. Multivariable Cox proportional hazards regression was used to estimate hazard ratios (HRs) and 95% confidence intervals (CIs) for the effects of IRA revascularization (in all patients) and CR (in patients with MVD) within risk-adjusted models. Covariates, including age, sex, body mass index, prior myocardial infarction, left main CAD, and diffuse CAD, were selected for adjustment based on clinical relevance and *p*-values less than 0.05 in univariable analyses to account for potential confounding factors [5, 7, 13, 19]. To further assess the robustness of our findings, we performed a sensitivity analysis including patients who died within 30 days post-surgery. Additionally, a multivariable Cox model, which incorporated all revascularization-related variables, was adjusted using a backward stepwise selection strategy to identify factors associated with the outcomes. All statistical analyses were performed using R software (version 4.3.1, R Foundation for Statistical Computing, Vienna, Austria). A two-tailed *p*-value of less than 0.05 was considered statistically significant.

## 3. Results

A total of 132 patients, with a median age of 63 years, were included in the final analysis, and 34% were female. Among these patients, 28 did not undergo IRA revascularization. Table 1 presents the characteristics of patients who did and did not receive IRA revascularization. Among the 84 patients with MVD, 53 achieved CR, while 31 had ICR. The characteristics of these two groups are summarized in Table 2.

**Table 1.** **Clinical characteristics by infarct artery revascularization status**.

Characteristics		IRA	No IRA	*p* value
		Revascularization	Revascularization	
		(n = 104)	(n = 28)	
Demographics				
	Age, y	63.0 (57.0–68.0)	61.5 (56.0–67.0)	0.560
	Female sex	36 (34.6)	10 (35.7)	0.914
Risk factors and history				
	Body mass index, kg/m^2^	24.7 (22.7–26.4)	24.6 (22.3–26.1)	0.557
	Diabetes mellitus	32 (30.8)	8 (28.6)	0.822
	Hypertension	63 (60.6)	15 (53.6)	0.503
	Stroke	13 (12.5)	3 (10.7)	1.000
	Prior or current smoking	62 (59.6)	12 (42.9)	0.113
	Prior MI	7 (6.7)	1 (3.6)	1.000
	LVEF, %	50.0 (41.0–55.0)	45.5 (39.0–55.8)	0.168
Diseased coronary vessels				
	Multi-vessel disease	71 (68.3)	13 (46.4)	0.033
	Left main disease	3 (3.8)	1 (3.6)	1.000
	IRA - LAD	83 (79.8)	21 (75)	0.581
	IRA - RCA	21 (20.2)	7 (25)	
	Totally occluded IRA	46 (44.2)	19 (67.9)	0.026
	Diffusely stenosed IRA	32 (30.8)	12 (42.9)	0.228
Location of rupture				
	Apical	54 (51.9)	16 (57.1)	0.623
	Anterior	21 (20.2)	5 (17.9)	0.783
	Posterior	29 (27.9)	7 (25.0)	0.761
Treatments				
	Preoperative IABP	36 (34.6)	12 (42.9)	0.421
	Preoperative PCI	35 (33.7)	1 (3.6)	0.002
	Time from MI to surgery, d	47.0 (37.0–69.0)	58.0 (39.0–71.0)	0.697
	CABG	92 (88.5)	13 (46.4)	<0.001
	Arterial graft	59 (56.7)	4 (14.3)	<0.001
	Postoperative IABP	5 (4.8)	0 (0)	0.584
Re-exploration for bleeding		7 (6.7)	1 (3.6)	1.000
Postoperative shunt		5 (4.8)	1 (3.6)	1.000

Data are presented as means ± standard deviations or median (interquartile range) or number (%). Abbreviations: CABG, coronary artery bypass grafting; IABP, intra-aortic balloon pump; IRA, infarct-related artery; LAD, left anterior descending (artery); LVEF, left ventricular ejection fraction; MI, myocardial infarction; PCI, percutaneous coronary intervention; RCA, right coronary artery.

**Table 2.** **Clinical characteristics by revascularization completeness**.

Characteristics		Complete Revascularization	Incomplete Revascularization	*p* value
		(n = 53)	(n = 31)	
Demographics				
	Age, y	63.0 (56.5–68.5)	62.0 (59.0–66.0)	0.707
	Female sex	18 (34.0)	10 (32.3)	0.873
Risk factors and history				
	Body mass index, kg/m^2^	24.0 (22.2–25.7)	24.2 (21.2–25.4)	0.502
	Diabetes mellitus	14 (26.4)	9 (29.0)	0.795
	Hypertension	30 (56.6)	21 (67.7)	0.313
	Stroke	7 (13.2)	5 (16.1)	0.753
	Prior or current smoking	32 (60.4)	17 (54.8)	0.619
	Prior MI	5 (9.4)	2 (6.5)	1.000
	LVEF, %	50.0 (41.0–55.5)	49.0 (39.0–58.0)	0.587
Diseased coronary vessels				
	Left main disease	1 (1.9)	4 (12.9)	0.060
	IRA - LAD	37 (69.8)	26 (83.9)	0.151
	IRA - RCA	16 (30.2)	5 (16.1)	
	Totally occluded IRA	23 (43.4)	21 (67.7)	0.031
	Diffusely stenosed IRA	17 (32.1)	14 (45.2)	0.230
Location of rupture				
	Apical	27 (50.9)	21 (67.7)	0.133
	Anterior	8 (15.1)	3 (9.7)	0.739
	Posterior	18 (34.0)	7 (22.6)	0.271
Treatments				
	Preoperative IABP	18 (34.0)	13 (41.9)	0.465
	Preoperative PCI	15 (28.3)	7 (22.6)	0.565
	Time from MI to repair, d	48.0 (36.0–69.0)	61.0 (42.3–70.8)	0.352
	CABG	51 (96.2)	28 (90.3)	0.353
	Arterial graft	34 (64.2)	13 (41.9)	0.048
	Revascularization of IRA	53 (100)	19 (61.3)	<0.001
	Revascularization of non-IRA	53 (100)	22 (71.0)	<0.001
	Postoperative IABP	2 (3.8)	2 (6.5)	0.624
Re-exploration for bleeding		3 (5.7)	2 (6.5)	1.000
Postoperative shunt		1 (1.9)	2 (6.5)	0.552

Data are presented as means ± standard deviations or median (interquartile range) or number (%).

### 3.1 IRA Revascularization vs. No IRA Revascularization

There were no significant differences in demographic characteristics or comorbidities between the groups with and without IRA revascularization. However, patients in the IRA revascularization group had a higher prevalence of MVD and a greater proportion who underwent PCI before surgical repair. Of these, 12 patients (11.5%) completed revascularization solely via PCI. In the no IRA revascularization group, all 13 patients (46.4%) with MVD underwent revascularization of non-IRA.

**Fig. 1.**
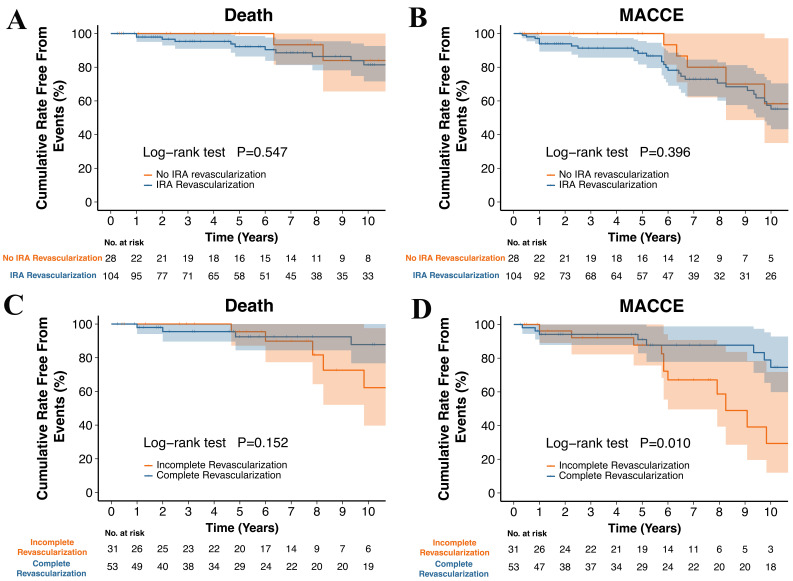
**Kaplan-Meier survival curves and MACCE incidence in patients with ventricular septal rupture undergoing different revascularization approaches**. (A) All-cause mortality, IRA revascularization vs. no IRA revascularization. (B) MACCE, IRA revascularization vs. no IRA revascularization. (C) All-cause mortality, CR vs. ICR in MVD patients. (D) MACCE, CR vs. ICR in MVD patients. Abbreviations: CR, complete revascularization; ICR, incomplete revascularization; MACCE, major adverse cardiovascular and cerebrovascular events; MVD, multi-vessel disease.

The mean follow-up period was 77.8 months (median 71.0 months). During this time, 24 patients died of all causes, and 45 experienced MACCE. Kaplan-Meier analysis showed 10-year cumulative survival rates of 81.4% in the IRA revascularization group and 84.0% in the no IRA revascularization group (*p *= 0.547). The corresponding rates of freedom from MACCE were 55.2% and 58.3%, respectively (*p* = 0.396), as shown in Fig. 1A,B. Adjusted Cox analysis (Table 3) revealed no significant association between IRA revascularization and long-term mortality (HR: 0.62; 95% CI: 0.22–1.79; *p* = 0.376) or MACCE (HR: 1.30; 95% CI: 0.52–3.27; *p* = 0.575). Sensitivity analysis, including early mortality patients, further supported these findings (**Supplementary Table 1**). Subgroup analysis showed similar outcomes between single-vessel and MVD patients, with interaction *p*-values of 0.967 for mortality and 0.343 for MACCE. No evidence of heterogeneity was observed across subgroups stratified by age, sex, and diabetes (**Supplementary Tables 2,3**). Furthermore, IRA revascularization performed via either CABG or PCI showed no significant differences in its impact on long-term mortality (HR: 0.59; 95% CI: 0.14–2.58; *p* = 0.483) or MACCE (HR: 0.73; 95% CI: 0.28–1.88; *p* = 0.515).

**Table 3.** **Impact of different revascularization strategies on long-term outcomes**.

Group		n (%)	Unadjusted		Adjusted	
			HR (95% CI)	*p* value	HR (95% CI)	*p* value
All-Cause Mortality						
All patients (n = 132)						
	No IRA Revascularization	5/28 (17.9)	Reference		Reference	
	IRA Revascularization	19/104 (18.3)	0.74 (0.27–2.01)	0.551	0.62 (0.22–1.79)	0.376
MVD patients (n = 84)						
	Incomplete Revascularization	7/31 (22.6)	Reference		Reference	
	Complete Revascularization	9/53 (17.0)	0.47 (0.16–1.35)	0.146	0.57 (0.17–1.85)	0.346
MACCE						
All patients (n = 132)						
	No IRA Revascularization	6/28 (21.4)	Reference		Reference	
	IRA Revascularization	39/104 (37.5)	1.45 (0.61–3.44)	0.401	1.30 (0.52–3.27)	0.575
MVD patients (n = 84)						
	Incomplete Revascularization	13/31 (41.9)	Reference		Reference	
	Complete Revascularization	15/53 (28.3)	0.37 (0.17–0.81)	0.013	0.26 (0.10–0.67)	0.005

Abbreviations: CI, confidence interval; HR, hazard ratio.

### 3.2 CR vs. ICR

No significant differences in demographic characteristics or comorbidities were observed between the CR and ICR groups. In the CR group, 2 patients did not undergo bypass surgery, while 3 patients in the ICR group also did not. Among these, 1 patient lacked suitable grafting sites, and 4 had already undergone PCI prior to surgery. Of the ICR patients, 19 (61.3%) had ICR of non-IRA, 11 (35.5%) did not undergo IRA revascularization, and 1 (3.2%) lacked revascularization of both IRA and non-IRA. Arterial grafts were used more frequently in the CR group than in the ICR group (64.2% vs. 41.9%, *p* = 0.048). The average number of coronary anastomoses was 2.27 ± 1.08, with significantly more anastomoses in the CR group (2.64 ± 1.08) compared to the ICR group (1.65 ± 1.14, *p* < 0.001).

**Fig. 2.**
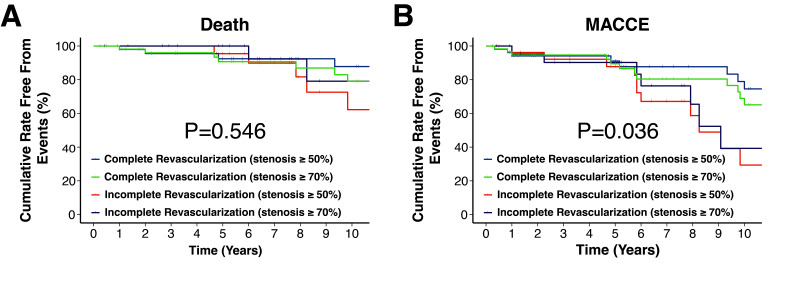
**Kaplan-Meier survival curves and MACCE incidence in patients with multi-vessel disease, where complete revascularization is defined as revascularization of vessels with stenosis ≥70%**. (A) All-cause mortality. (B) MACCE.

The mean follow-up period was 79.0 months (median 69.0 months). During this time, 10 cases of all-cause mortality and 28 cases of MACCE were observed. Kaplan-Meier analysis showed 10-year cumulative survival rates of 87.8% for the CR group and 62.2% for the ICR group (*p* = 0.152), with corresponding rates of freedom from MACCE of 74.5% and 29.4% (*p* = 0.010), as illustrated in Fig. 1C,D. Adjusted Cox regression analysis (Table 3) revealed no statistically significant difference in survival rates between the CR and ICR groups (HR: 0.57; 95% CI: 0.17–1.85; *p* = 0.346). However, the incidence of MACCE was significantly lower in the CR group (HR: 0.26; 95% CI: 0.10–0.67; *p* = 0.005). This effect remained consistent when CR was defined as revascularization of vessels with more than 70% stenosis (Fig. 2A,B) and across other subgroups (**Supplementary Tables 4,5**). Moreover, among ICR patients, revascularization of non-IRA, compared to revascularization of the IRA alone, showed a trend toward reducing long-term MACCE (HR: 0.256; 95% CI: 0.055–1.194; *p* = 0.083).

Finally, we assessed the relationship between revascularization-related variables and long-term survival based on complete cases. Univariate analysis identified three variables—diffuse CAD (HR 3.20; 95% CI: 1.31–7.83, *p* = 0.011), left main disease (HR 3.62; 95% CI: 1.05–12.56, *p* = 0.042), and CR (HR 0.39; 95% CI: 0.17–0.92, *p *= 0.031)—as being associated with all-cause mortality. However, after stepwise variable selection, only diffuse CAD was retained.

## 4. Discussion

This single-center observational study, which included a relatively large cohort of VSR patients, aimed to assess the impact of revascularization completeness on long-term clinical outcomes following surgical repair. The results demonstrated that IRA revascularization did not significantly improve long-term survival or reduce the incidence of MACCE. However, among patients with MVD, CR was associated with a significantly lower incidence of MACCE compared to ICR, although it did not have a significant impact on survival rates.

To date, reports on the effectiveness of revascularization in VSR patients have been limited, likely due to the rarity of the condition, the small number of cases managed annually by most centers, and variations in treatment preferences [20]. A meta-analysis by Horan *et al*. [3] found that CABG was performed in 52% of patients, but revascularization of the infarcted area was achieved in only 54% of those cases. One major reason for avoiding IRA revascularization is the assumption that the infarcted territory associated with VSR has already concluded, rendering blood flow restoration of limited benefit [2, 3, 6]. Additionally, procedural risks, including increased surgical complexity and the bleeding risks associated with antiplatelet therapy, may outweigh the potential advantages [2, 6]. However, these risks are not uniform and may depend on factors such as the timing of intervention, surgical technique, and patient condition.

Acute-phase IRA revascularization may provide the greatest benefit. In a cohort of 102 patients with a median of 2 days between VSR and surgery, Lundblad *et al*. [5] observed that both IRA revascularization and CR were associated with improved 30-day survival. This improvement may result from enhanced perfusion of the ischemic border zone and better control of ventricular arrhythmias, even if there is no direct benefit to the infarcted myocardium. Similar findings were reported by Dogra *et al*. [21], who noted that early thrombolysis improved postoperative survival. Conversely, a recent multicenter registry study by Giblett *et al*. [22], which had a median interval of 9 days from AMI to surgery, found an association between PCI of the IRA and in-hospital mortality. These findings align with evidence suggesting that the benefits of IRA revascularization in the acute phase diminish over time [7, 8]. It is worth noting that cardiogenic shock—commonly treated with revascularization in AMI patients—is less relevant in VSR cases, as it primarily results from acute left-to-right shunting [2]. In such instances, treatment focuses on shunt reduction or defect closure, warranting cautious consideration of IRA revascularization several days post-AMI and VSR onset.

As the time from AMI to surgery increases, the risks and benefits of revascularization shift. At our center, surgery is typically delayed to allow for patient stabilization, reducing surgical risks and improving postoperative recovery. Consequently, revascularization in this study was performed later, with no observed impact on all-cause mortality or MACCE. This finding may partially reflect limited statistical power but also underscores the limited utility of revascularization in infarcted regions with lost functional capacity [22]. Nevertheless, some studies have reported improved long-term outcomes with IRA revascularization despite its lack of effect on early mortality [22, 23]. Overall, there is insufficient evidence to confirm that IRA revascularization has a detrimental effect, although it may not always be beneficial. Our study demonstrates that IRA revascularization during delayed repair is feasible and adds to the knowledge gap regarding the effects of timing and patient characteristics on intervention outcomes. We routinely perform IRA revascularization, except in cases where target segments are within ventricular aneurysms, the ventriculotomy suture line, or where diffuse coronary stenosis or anatomical factors make the procedure unfeasible.

We also found that cardiopulmonary bypass time was not significantly associated with survival. In studies by Held and Takahashi, longer CPB times were linked to early mortality [12, 24]. Interestingly, they also found that ICR—rather than CABG itself—was identified as an independent risk factor. This may be explained by non-survivors having shorter intervals between VSR onset and surgery [11, 25], poorer ventricular function [26, 27], and worse preoperative hemodynamics [28], all of which likely contribute to difficulties in weaning from CPB, leading to longer surgeries and higher postoperative mortality, particularly in urgent or early operations [10, 26, 29, 30, 31].

The extent and severity of CAD have been reported to correlate with poor prognosis in VSR patients [22]. Jeppsson *et al*. [13] identified the number of anastomoses as an independent predictor of late mortality, with each additional anastomosis increasing the risk by 1.5 times. This likely reflects the greater disease burden in patients with more extensive CAD, leading to worse outcomes, a finding further supported by Giblett *et al*. [22]. However, revascularization may overcome the adverse effects of extensive CAD [4]. Our study showed that the 10-year survival rate of MVD patients who received CR was comparable to that of patients with single-vessel disease who underwent IRA revascularization (94.4% vs. 87.8%, *p* = 0.47), suggesting that the completeness of revascularization may have a greater impact on survival outcomes in MVD patients than the extent of the lesion. Similarly, Muehrcke* et al*. [4] reported that patients with two- or three-vessel disease who underwent CABG had significantly better long-term survival compared to those who did not, despite similar baseline characteristics. In our study, while long-term mortality rates were similar between the CR and ICR groups, the incidence of MACCE was significantly lower in the CR group. This finding suggests that CR reduces composite endpoints, including readmissions for heart failure, myocardial infarction, repeat revascularization, and stroke. The benefits of revascularizing non-culprit vessels may be attributed to enhanced myocardial collateral circulation, which promotes recovery and reduces the long-term risk of additional myocardial ischemia caused by progressive atherosclerosis and luminal stenosis [4, 12]. Among ICR patients, those who underwent revascularization of non-IRA experienced a lower incidence of MACCE compared to those who had revascularization limited to the IRA, further reinforcing this mechanism. Therefore, when performing delayed repair in VSR patients with MVD, CR should be prioritized, or, if not feasible, significant stenoses in non-IRA should be addressed to optimize outcomes.

## 5. Limitations

This study has several limitations that should be acknowledged. First, as a retrospective study, it is inherently subject to biases and unmeasured confounders that could influence the results and limit the generalizability of the findings. Surgical details were obtained from medical records, and in some cases, the reasons for not revascularizing the IRA or non-IRA were incomplete or unavailable, preventing a comprehensive analysis of these factors, which may have been relevant to the outcomes. Additionally, since most patients in this study underwent delayed surgical repair, the findings primarily reflect a selectively stable cohort and may not be directly applicable to patients requiring early surgery. While differences in revascularization timing have been analyzed in the discussion section, this limitation should still be considered.

Second, the relatively small sample size may have limited the study’s statistical power, even though it represents one of the largest cohorts to date examining revascularization outcomes in VSR patients. Moreover, significant variability in VSR management strategies across centers, as documented in prior investigations, may have contributed to differences in outcomes. Larger, multicenter studies are essential to validate these findings and address these variations.

Third, due to the limited number of events, patients who died early were excluded, restricting the evaluation for the impact of early revascularization on VSR patients. However, sensitivity analyses that included these patients demonstrated consistent trends, thereby reinforcing the robustness of our conclusions. Despite these limitations, as the approach of stabilizing patients and delaying surgery gains recognition as a viable strategy, the insights provided by this study may serve as a valuable reference for clinical decision-making regarding the timing and strategy of revascularization in VSR patients.

## 6. Conclusions

In patients undergoing surgical repair for VSR, revascularization of the IRA did not improve long-term survival or reduce the incidence of MACCE compared to those without IRA revascularization. However, CR appears to lower the long-term risk of MACCE in patients with MVD, although it did not significantly affect mortality. These findings warrant validation in larger prospective studies.

## Availability of Data and Materials

The datasets used and/or analyzed during the current study are available from the corresponding author on reasonable request.

## Supplementary Material

Supplementary material associated with this article can be found, in the online version, at https://doi.org/10.31083/RCM27049.
