# Supplementary material for: Impact of Completeness of Revascularization on Long-Term Outcomes in Patients With Post-Infarction Ventricular Septal Rupture
Source: Rev Cardiovasc Med. 2025 Jun 16;26(6):27049. doi: 10.31083/RCM27049 (PMC12230848; doi:10.31083/RCM27049)
Supplement: Supplementary file 1 [file 2153-8174-26-6-27049-s1.docx]

**SUPPLEMENTAL MATERIAL**

**Title:** **Impact of Completeness of Revascularization on Long-Term Outcomes in Patients with Post-Infarction Ventricular Septal Rupture**

**Supplementary Tables**

Supplementary Table 1. **Sensitivity Analysis for Long-term Outcomes Including Patients with Early Mortality**

Supplementary Table 2. **Cumulative incidence of mortality according to IRA revascularization, stratified by age, sex, diabetes, and multivessel disease**

Supplementary Table 3. **Cumulative incidence of MACCE according to IRA revascularization, stratified by age, sex, diabetes, and multivessel disease**

Supplementary Table 4. **Cumulative incidence of mortality according to completeness of revascularization, stratified by age, sex, and diabetes**

Supplementary Table 5. **Cumulative incidence of MACCE according to completeness of revascularization, stratified by age, sex, and diabetes**

**Supplementary Table 1. Sensitivity Analysis for Long-term Outcomes Including Patients with Early Mortality**

|  |  | **Unadjusted** | | **Adjusted** | |
| --- | --- | --- | --- | --- | --- |
| **Group** | **n (%)** | **HR (95% CI)** | ***P* Value** | **HR (95% CI)** | ***P* Value** |
| **All-Cause Mortality** |  |  |  |  |  |
| All patients (n=141) |  |  |  |  |  |
| No IRA Revascularization | 9/32 (28.1) | Reference |  | Reference |  |
| IRA Revascularization | 24/109 (22.0) | 0.58 (0.26-1.27) | 0.171 | 0.66 (0.23-1.86) | 0.426 |
| MVD patients (n=90) |  |  |  |  |  |
| Incomplete Revascularization | 10/34 (29.4) | Reference |  | Reference |  |
| Complete Revascularization | 12/56 (21.4) | 0.70 (0.28-1.76) | 0.445 | 0.66 (0.24-1.82) | 0.421 |
| **MACCE** |  |  |  |  |  |
| All patients (n=141) |  |  |  |  |  |
| No IRA Revascularization | 10/32 (31.3) | Reference |  | Reference |  |
| IRA Revascularization | 44/109 (40.4) | 1.03 (0.51-2.05) | 0.940 | 1.15 (0.46-2.88) | 0.764 |
| MVD patients (n=90) |  |  |  |  |  |
| Incomplete Revascularization | 16/34 (47.1) | Reference |  | Reference |  |
| Complete Revascularization | 18/56 (32.1) | 0.54 (0.26-1.13) | 0.101 | 0.42 (0.18-0.94) | 0.036 |

Abbreviations: CI, confidence interval; HR, hazard ratio; IRA, infarct-related artery; MACCE, major adverse cardiovascular and cerebrovascular events; MVD, multi-vessel disease

**Supplementary Table 2. Cumulative incidence of mortality according to IRA revascularization, stratified by age, sex, diabetes, and multivessel disease**

| Subgroup | Number of patients | | Hazard ratio (95% CI) | *P* Value | Interaction *P* Value |
| --- | --- | --- | --- | --- | --- |
|  | IRA Revascularization (n=104) | No IRA Revascularization (n=28) |  |  |  |
| Overall | 104 | 28 | 0.736 (0.269-2.014) | 0.551 |  |
| Age, years |  |  |  |  |  |
| <65 | 59 | 18 | 0.541 (0.139-2.109) | 0.377 | 0.916 |
| ≥65 | 45 | 10 | 1.157 (0.248-5.400) | 0.853 |  |
| Sex |  |  |  |  |  |
| Male | 68 | 18 | 0.611 (0.196-1.909) | 0.611 | 0.507 |
| Female | 36 | 10 | 1.421 (0.159-12.719) | 1.421 |  |
| Diabetes mellitus |  |  |  |  |  |
| Yes | 32 | 8 | 1.290 (0.251-6.634) | 0.760 | 0.915 |
| No | 72 | 20 | 0.794 (0.215-2.933) | 0.729 |  |
| Multi-vessel disease |  |  |  |  |  |
| Yes | 71 | 13 | 0.691 (0.192-2.485) | 0.572 | 0.967 |
| No | 33 | 15 | 0.734 (0.137-3.925) | 0.718 |  |

Abbreviations: IRA, infarct-related artery

**Supplementary Table 3. Cumulative incidence of MACCE according to IRA revascularization, stratified by age, sex, diabetes, and multivessel disease**

| Subgroup | Number of patients | | Hazard ratio (95% CI) | *P* Value | Interaction *P* Value |
| --- | --- | --- | --- | --- | --- |
|  | IRA Revascularization (n=104) | No IRA Revascularization (n=28) |  |  |  |
| Overall | 104 | 28 | 1.449 (0.609-3.443) | 0.401 |  |
| Age, years |  |  |  |  |  |
| <65 | 59 | 18 | 1.523 (0.449-5.164) | 0.499 | 0.799 |
| ≥65 | 45 | 10 | 1.363 (0.393-4.723) | 0.626 |  |
| Sex |  |  |  |  |  |
| Male | 68 | 18 | 1.198 (0.411-3.491) | 0.741 | 0.690 |
| Female | 36 | 10 | 2.099 (0.469-9.394) | 0.332 |  |
| Diabetes mellitus |  |  |  |  |  |
| Yes | 32 | 8 | 4.402 (0.565-34.280) | 0.157 | 0.618 |
| No | 72 | 20 | 1.292 (0.443-3.766) | 0.639 |  |
| Multi-vessel disease |  |  |  |  |  |
| Yes | 71 | 13 | 0.985 (0.339-2.867) | 0.978 | 0.343 |
| No | 33 | 15 | 2.489 (0.550-11.258) | 0.236 |  |

Abbreviations: IRA, infarct-related artery; MACCE, major adverse cardiovascular and cerebrovascular events

**Supplementary Table 4. Cumulative incidence of mortality according to completeness of revascularization, stratified by age, sex, and diabetes**

| Subgroup | Number of patients | | Hazard ratio (95% CI) | *P* Value | Interaction *P* Value |
| --- | --- | --- | --- | --- | --- |
|  | Complete Revascularization  (n = 53) | Incomplete Revascularization  (n = 31) |  |  |  |
| Overall | 53 | 31 | 0.469 (0.163-1.351) | 0.146 |  |
| Age, years |  |  |  |  |  |
| <65 | 30 | 19 | 0.285 (0.062-1.307) | 0.106 | 0.652 |
| ≥65 | 23 | 12 | 0.731 (0.163-3.282) | 0.682 |  |
| Sex |  |  |  |  |  |
| Male | 35 | 21 | 0.722 (0.216-2.763) | 0.691 | 0.351 |
| Female | 18 | 10 | 0.209 (0.021-2.110) | 0.185 |  |
| Diabetes mellitus |  |  |  |  |  |
| Yes | 14 | 9 | 1.570 (0.141-17.461) | 0.714 | 0.615 |
| No | 39 | 22 | 0.459 (0.131-1.609) | 0.224 |  |

**Supplementary Table 5. Cumulative incidence of MACCE according to completeness of revascularization, stratified by age, sex, and diabetes**

| Subgroup | Number of patients | | Hazard ratio (95% CI) | *P* Value | Interaction *P* Value |
| --- | --- | --- | --- | --- | --- |
|  | Complete Revascularization  (n = 53) | Incomplete Revascularization  (n = 31) |  |  |  |
| Overall | 53 | 31 | 0.366 (0.166-0.811) | 0.013 |  |
| Age, years |  |  |  |  |  |
| <65 | 30 | 19 | 0.316 (0.103-0.973) | 0.045 | 0.847 |
| ≥65 | 23 | 12 | 0.492 (0.162-1.492) | 0.210 |  |
| Sex |  |  |  |  |  |
| Male | 35 | 21 | 0.481 (0.173-1.334) | 0.160 | 0.152 |
| Female | 18 | 10 | 0.197 (0.037-1.055) | 0.058 |  |
| Diabetes mellitus |  |  |  |  |  |
| Yes | 14 | 9 | 0.377 (0.080-1.781) | 0.218 | 0.797 |
| No | 39 | 22 | 0.422 (0.158-1.131) | 0.086 |  |
